# (1*S*,2*E*,6*R*,7a*R*)-1,6-Dihy­droxy-2-(4-nitro­benzyl­idene)-2,3,5,6,7,7a-hexa­hydro-1*H*-pyrrolizin-3-one

**DOI:** 10.1107/S1600536812018235

**Published:** 2012-04-28

**Authors:** F. L. Oliveira, K. R. L. Freire, R. Aparicio, F. Coelho

**Affiliations:** a Laboratory of Structural Biology and Crystallography, Institute of Chemistry, University of Campinas, CP6154, CEP13083-970, Campinas, SP, Brazil; b Laboratory of Synthesis of Natural Products and Drugs, Institute of Chemistry, University of Campinas, CP6154, CEP13083-970, Campinas, SP, Brazil

## Abstract

The crystal structure of the title compound, C_14_H_14_N_2_O_5_, contains two distinct conformers in the asymmetric unit. The compound has three defined stereocenters, two of them contiguous, and a C=C double bond with an *E* conformation. The stereocenters exhibit the same chirality in both conformers, with significant differences in the conformation of the five-membered rings of the pyrrolizine unit (both either in a twist or in an envelope form) and in the dihedral angles between the corresponding mean planes and the benzene rings. A prominent feature is a change from almost coplanar rings in one conformer to a new conformation in the second conformer, in which the mean plane of a five-membered ring is almost perpendicular to the benzene ring, with a dihedral angle 87.19 (8)°; the corresponding angle in the first conformer is 14.02 (10)°. In the crystal, molecules are linked by O—H⋯O and C—H⋯O hydrogen bonds. Crystallographic data were essential to confirm the configuration of the double bond, which was unclear from the available two-dimensional NMR data. In addition, reliable Flack and Hooft parameters were obtained, allowing for the correct absolute structure to be determined.

## Related literature

For the preparation of the title compound, see: Freire *et al.* (2011 ▶). For the use of this type of compound as LFA-1 (Lymphocyte Function-Associated Anti­gen-1) inhibitors, see: Baumann (2007 ▶). For related structures, see: Oliveira *et al.* (2012*a* ▶,*b* ▶).
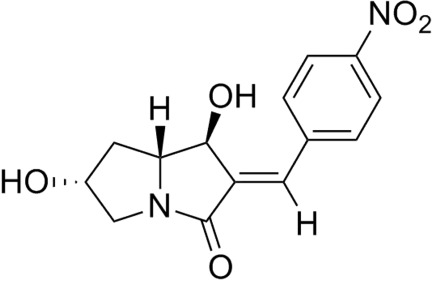


## Experimental

### 

#### Crystal data

C_14_H_14_N_2_O_5_
*M* *_r_* = 290.27
Monoclinic, 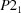

*a* = 6.8289 (6) Å
*b* = 7.0433 (6) Å
*c* = 26.618 (3) Å
β = 92.335 (4)°
*V* = 1279.2 (2) Å^3^
*Z* = 4
Cu *K*α radiation
μ = 0.98 mm^−1^
*T* = 100 K
0.39 × 0.23 × 0.06 mm

#### Data collection

Bruker Kappa APEXII DUO diffractometer
Absorption correction: numerical (*SADABS*; Bruker, 2010 ▶) *T*_min_ = 0.844, *T*_max_ = 1.000
41060 measured reflections
4038 independent reflections
3986 reflections with *I* > 2σ(*I*)
*R*_int_ = 0.037

#### Refinement

*R*[*F* ^2^ > 2σ(*F* ^2^)] = 0.025
*wR*(*F* ^2^) = 0.067
*S* = 1.01
4038 reflections
383 parameters
1 restraint
H-atom parameters constrained
Δρ_max_ = 0.15 e Å^−3^
Δρ_min_ = −0.16 e Å^−3^
Absolute structure: Flack (1983 ▶) and Hooft *et al.* (2008 ▶); Hooft parameter = 0.04(4), 1539 Bijvoet pairs
Flack parameter: 0.03 (11)

### 

Data collection: *APEX2* (Bruker, 2010 ▶); cell refinement: *SAINT* (Bruker, 2010 ▶); data reduction: *SAINT*; program(s) used to solve structure: *SHELXS97* (Sheldrick, 2008 ▶); program(s) used to refine structure: *SHELXL97* (Sheldrick, 2008 ▶); molecular graphics: *PLATON* (Spek, 2009 ▶); software used to prepare material for publication: *publCIF* (Westrip, 2010 ▶).

## Supplementary Material

Crystal structure: contains datablock(s) I, global. DOI: 10.1107/S1600536812018235/pv2525sup1.cif

Structure factors: contains datablock(s) I. DOI: 10.1107/S1600536812018235/pv2525Isup2.hkl

Supplementary material file. DOI: 10.1107/S1600536812018235/pv2525Isup3.cml

Additional supplementary materials:  crystallographic information; 3D view; checkCIF report

## Figures and Tables

**Table 1:** Hydrogen-bond geometry (Å, °)

*D*—H⋯*A*	*D*—H	H⋯*A*	*D*⋯*A*	*D*—H⋯*A*
O3—H3⋯O1′^i^	0.82	1.93	2.7303 (14)	166
O1—H1*A*⋯O2^ii^	0.82	1.89	2.6993 (15)	169
O3′—H3′⋯O3^iii^	0.82	1.95	2.7585 (15)	171
O1′—H1*A*′⋯O3′^iv^	0.82	1.93	2.7451 (15)	174
C1′—H1′⋯O2′^ii^	0.98	2.52	3.3655 (17)	144
C1′—H1′⋯O5′^v^	0.98	2.55	3.3242 (19)	136
C10—H10⋯O1^vi^	0.93	2.54	3.126 (2)	121
C12′—H12′⋯O3′^vii^	0.93	2.57	3.5014 (18)	174
C13—H13⋯O4^i^	0.93	2.51	3.2644 (19)	138
C13′—H13′⋯O5′^i^	0.93	2.51	3.1160 (18)	123

Symmetry codes: (i) 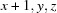; (ii) 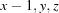; (iii) 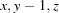; (iv) 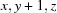; (v) 
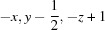
; (vi) 
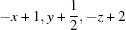
; (vii) 
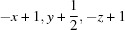
.

**Fig. 1.**
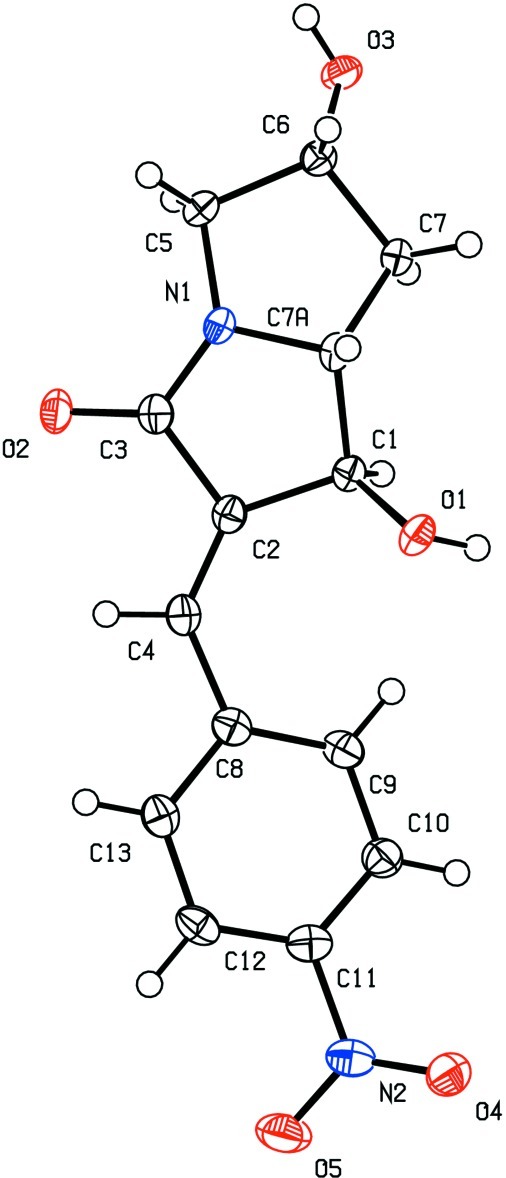
A view of molecule 1 with the atom numbering scheme. Displacement ellipsoids are drawn at the 50% probability level. H atoms are presented as small spheres of arbitrary radius.

**Fig. 2.**
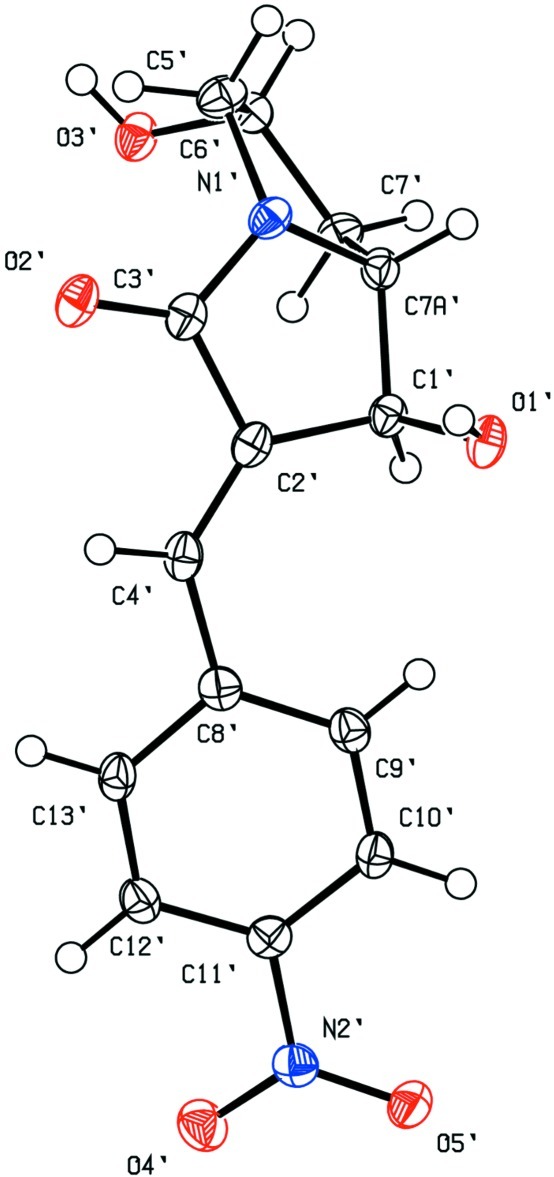
A view of molecule 2 with the atom numbering scheme. Displacement ellipsoids are drawn at the 50% probability level. H atoms are presented as small spheres of arbitrary radius.

**Fig. 3.**
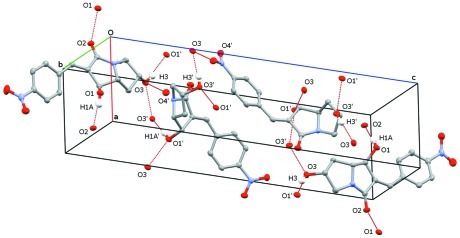
A view of the hydrogen bonding interactions (dotted lines) in the crystal structure of the title compound. H atoms non-participating in hydrogen-bonding were omitted for clarity.

## supplementary crystallographic information

### Comment

The title compound, a new asymmetric benzyl-pyrrolizidinone, has been prepared from a Morita-Baylis-Hillman adduct using a straight forward synthetic sequence developed in our laboratory (Freire *et al.*, 2011). It can be used as a prototype to design new mediators of the activity of LFA-1 (lymphocyte function-associated antigen 1), with potential applications in the treatment of autoimmune diseases and as anti-inflammatory drugs (Baumann, 2007). The title compound has three defined stereocenters, two of them contiguous, and a double bond with E configuration, an information which was not clear from two-dimensional-NOESY NMR studies. A crystal structure determination of the titlte compound has allowed us to establish its correct configuration, which is reported in this article.

In the crystal structure of the title compound, an asymmetric unit contains two distinct conformers, molecule 1 (Fig. 1) and molecule 2 (Fig. 2), whose stereocenters exhibit the same chirality. In molecule 1, the five membered rings N1/C3/C2/C1/C7A and N1/C5/C6/C7/C7A of the pyrrolizine moiety exhibit C1- and C7-envelope conformations, respectively, with C1 and C7 atoms displaced from the mean-planes formed by the remaining rings atoms by 0.3194 (14) and 0.592 (2) Å, respectively. The mean planes of these rings have a dihedral angle of 23.47 (10)°, and are almost coplanar to the benzene ring, with which they form dihedral angles of 24.52 (9)° and 14.02 (10)°, respectively. The molecule 2 exhibits the rings N1'/C7A'/C1'/C2'/C3' and N1'/C7A'/C7'/C6'/C5' in a twisted conformation on C7A'-C1' and N1'-C7A', respectively, with a dihedral angle between their mean planes equal to 58.07 (8)°. The dihedral angle between the mean planes of the ring N1'/C7A'/C1'/C2'/C3' and the benzene ring is 34.75 (7)°. The ring N1'/C7A'/C7'/C6'/C5' is almost perpendicular to the latter with a corresponding angle equal to 87.19 (8)°. The atom C1' lies 0.3200 (14) Å out of the plane formed by the rest of the ring atoms (N1'/C7A'/C2'/C3'). The corresponding measurement for the atom C7A' in relation to the mean plane formed by the other atoms in the ring (N1'/C7'/C6'/C5') is 0.4771 (13) Å. In the crystal, the molecules are held together by intermolecular hydrogen bonds (Tab. 1 and Fig. 3).

### Experimental

The title compound was prepared by a synthetic sequence recently described in the literature (Freire *et al.*, 2011) and purified by flash silica gel column chromatography (CH_2_Cl_2_ : MeOH – solvent gradient: 100:0 to 95:05) to afford 0.07 g (as a white solid) in 83% yield. It was then recrystallized using the liquid-vapor saturation method, dissolved in ethanol and crystallized subject to the vapor pressure of a second less polar liquid (ethyl ether), in a closed camera, providing the slow formation of crystals.

### Refinement

The calculated Flack parameter was F = 0.03 (11) (Flack, 1983). Analysis of the absolute structure was also performed using likelihood methods (Hooft *et al.*, 2008) as implemented in *PLATON* (Spek, 2009). The resulting value for the Hooft parameter was *y* = 0.04 (4), with a calculated probability for an inverted structure equal to 1x10^-109^. These results unequivocally indicate that the absolute structure has been correctly assigned. All H atoms were placed in calculated positions with O—H = 0.82 Å and C—H = 0.93, 0.97 and 0.98 Å for aryl, methylene and methyne H-atoms, respectively, and refined in the riding model approximation with *U*_iso_(H) = 1.5 *U*_eq_(O) or 1.2 *U*_eq_(C).

### Crystal data

**Table:**

C_14_H_14_N_2_O_5_	*F*(000) = 608
*M**_r_* = 290.27	*D*_x_ = 1.507 Mg m^−^^3^
Monoclinic, *P*2_1_	Cu *K*α radiation, λ = 1.54178 Å
*a* = 6.8289 (6) Å	Cell parameters from 4038 reflections
*b* = 7.0433 (6) Å	θ = 1.7–67.8°
*c* = 26.618 (3) Å	µ = 0.98 mm^−^^1^
β = 92.335 (4)°	*T* = 100 K
*V* = 1279.2 (2) Å^3^	Plate, orange
*Z* = 4	0.39 × 0.23 × 0.06 mm

### Data collection

**Table:**

Bruker Kappa APEXII DUO diffractometer	4038 independent reflections
Radiation source: fine-focus sealed tube	3986 reflections with *I* > 2σ(*I*)
Graphite monochromator	*R*_int_ = 0.037
Bruker APEX CCD area–detector scans	θ_max_ = 67.8°, θ_min_ = 1.7°
Absorption correction: numerical (*SADABS*; Bruker, 2010)	*h* = −8→8
*T*_min_ = 0.844, *T*_max_ = 1.000	*k* = −8→6
41060 measured reflections	*l* = −31→31

### Refinement

**Table:**

Refinement on *F*^2^	Secondary atom site location: difference Fourier map
Least-squares matrix: full	Hydrogen site location: inferred from neighbouring sites
*R*[*F*^2^ > 2σ(*F*^2^)] = 0.025	H-atom parameters constrained
*wR*(*F*^2^) = 0.067	*w* = 1/[σ^2^(*F*_o_^2^) + (0.0419*P*)^2^ + 0.3034*P*] where *P* = (*F*_o_^2^ + 2*F*_c_^2^)/3
*S* = 1.01	(Δ/σ)_max_ = 0.001
4038 reflections	Δρ_max_ = 0.15 e Å^−^^3^
383 parameters	Δρ_min_ = −0.16 e Å^−^^3^
1 restraint	Absolute structure: Flack (1983) and Hooft *et al.* (2008); Hooft parameter = 0.04(4), 1539 Bijvoet pairs
Primary atom site location: structure-invariant direct methods	Flack parameter: 0.03 (11)

### Special details

**Table:**

Experimental. [α]D^20^ + 28° (c 2, MeOH); IR (Film, ν_max_): 3308, 2974, 2924, 2864, 1699, 1671, 1644, 1596, 1523, 1513, 1435, 1381, 1346, 1314, 1264, 1244, 1220, 1202, 1133, 1104, 1075, 1055 cm^-1^; ^1^H NMR (400 MHz, CD_3_OD) δ 1.49 (ddd, *J* = 13.2, 8.3, 5.1 Hz, 1H, H-7 A); 2.49 (ddd, *J* = 13.3, 7.4, 6.2 Hz, 1H, H-7B); 3.38 (dd, *J* = 12.4, 5.8 Hz, 1H, H-5 A); 3.68 (dd, *J* = 12.4, 3.0 Hz, 1H, H-5B); 3.80 (td, *J* = 8.1, 2.4 Hz, 1H, H-7 C); 4.56 (qd, *J* = 5.8, 3.3 Hz 1H, H-6); 5.01 (t, *J* = 2.4 Hz, 1H, H-1); 7.45 (d, *J* = 2.3 Hz, 1H, H-4); 8.01 (d, *J* = 8.8 Hz, 2H, Ar); 8.26 (d, *J* = 8.9 Hz, 2H, Ar); ^13^C NMR (62.5 MHz, CD_3_CN) 39.2; 53.3; 68.4; 71.5; 72.5; 124.3; 132.4; 133.2; 140.5; 141.8; 148.6; 170.9; HRMS (ESI-TOF) *m/z* Calc. for C_14_H_15_N_2_O_5_ [*M* + H]^+^: 291.0981. Found 291.0989.
Geometry. All e.s.d.'s (except the e.s.d. in the dihedral angle between two l.s. planes) are estimated using the full covariance matrix. The cell e.s.d.'s are taken into account individually in the estimation of e.s.d.'s in distances, angles and torsion angles; correlations between e.s.d.'s in cell parameters are only used when they are defined by crystal symmetry. An approximate (isotropic) treatment of cell e.s.d.'s is used for estimating e.s.d.'s involving l.s. planes.
Refinement. Refinement of *F*^2^ against ALL reflections. The weighted *R*-factor *wR* and goodness of fit *S* are based on *F*^2^, conventional *R*-factors *R* are based on *F*, with *F* set to zero for negative *F*^2^. The threshold expression of *F*^2^ > σ(*F*^2^) is used only for calculating *R*-factors(gt) *etc*. and is not relevant to the choice of reflections for refinement. *R*-factors based on *F*^2^ are statistically about twice as large as those based on *F*, and *R*- factors based on ALL data will be even larger.

### Fractional atomic coordinates and isotropic or equivalent isotropic displacement parameters (Å^2^)

**Table:**

	*x*	*y*	*z*	*U*_iso_*/*U*_eq_	
O4	0.39324 (16)	0.4194 (2)	1.13861 (4)	0.0321 (3)	
O3	1.01737 (15)	0.58119 (18)	0.71964 (4)	0.0225 (2)	
H3	1.1038	0.5564	0.7002	0.034*	
O5	0.64579 (19)	0.4992 (2)	1.18584 (4)	0.0403 (3)	
O2	1.35576 (15)	0.51910 (18)	0.90371 (4)	0.0242 (3)	
O1	0.69151 (14)	0.31660 (18)	0.90951 (4)	0.0229 (2)	
H1A	0.5821	0.3645	0.9071	0.034*	
O4'	−0.02923 (16)	0.27989 (18)	0.37824 (4)	0.0276 (3)	
O3'	0.64995 (14)	−0.31144 (17)	0.68320 (4)	0.0213 (2)	
H3'	0.7590	−0.3523	0.6914	0.032*	
O5'	−0.20844 (14)	0.44237 (19)	0.42810 (4)	0.0253 (2)	
O2'	0.89538 (14)	0.19376 (18)	0.63315 (4)	0.0246 (2)	
O1'	0.32974 (14)	0.45702 (17)	0.66785 (4)	0.0208 (2)	
H1A'	0.4302	0.5197	0.6722	0.031*	
N1	1.11842 (16)	0.4299 (2)	0.84513 (4)	0.0190 (3)	
N2'	−0.05497 (17)	0.3612 (2)	0.41818 (5)	0.0200 (3)	
C8'	0.3952 (2)	0.3245 (2)	0.53145 (5)	0.0186 (3)	
N1'	0.67054 (17)	0.15506 (19)	0.69496 (5)	0.0182 (3)	
N2	0.5662 (2)	0.4640 (2)	1.14448 (5)	0.0268 (3)	
C11	0.6877 (2)	0.4734 (2)	1.10041 (6)	0.0230 (3)	
C10	0.6000 (2)	0.5089 (3)	1.05377 (6)	0.0243 (3)	
H10	0.4657	0.5303	1.0504	0.029*	
C9	0.7149 (2)	0.5122 (3)	1.01205 (6)	0.0231 (3)	
H9	0.6577	0.5391	0.9805	0.028*	
C8	0.9165 (2)	0.4754 (2)	1.01693 (5)	0.0204 (3)	
C4	1.0493 (2)	0.4734 (2)	0.97498 (5)	0.0203 (3)	
H4	1.1813	0.4820	0.9848	0.024*	
C2	1.0161 (2)	0.4615 (2)	0.92537 (5)	0.0182 (3)	
C1	0.83077 (19)	0.4455 (2)	0.89178 (5)	0.0181 (3)	
H1	0.7706	0.5712	0.8877	0.022*	
C7A	0.9103 (2)	0.3801 (2)	0.84114 (5)	0.0184 (3)	
H7C	0.8963	0.2421	0.8379	0.022*	
C7	0.84455 (19)	0.4745 (3)	0.79203 (5)	0.0200 (3)	
H7A	0.8157	0.6079	0.7970	0.024*	
H7B	0.7298	0.4122	0.7770	0.024*	
C6	1.0236 (2)	0.4485 (3)	0.75953 (5)	0.0197 (3)	
H6	1.0251	0.3191	0.7460	0.024*	
C12	0.8871 (2)	0.4445 (3)	1.10728 (6)	0.0242 (3)	
H12	0.9440	0.4252	1.1392	0.029*	
C13	0.9999 (2)	0.4452 (3)	1.06502 (6)	0.0224 (3)	
H13	1.1343	0.4251	1.0689	0.027*	
C5	1.2005 (2)	0.4771 (3)	0.79686 (5)	0.0219 (3)	
H5A	1.3075	0.3927	0.7893	0.026*	
H5B	1.2468	0.6073	0.7964	0.026*	
C3	1.1863 (2)	0.4727 (2)	0.89175 (5)	0.0187 (3)	
C11'	0.1040 (2)	0.3571 (2)	0.45687 (6)	0.0184 (3)	
C10'	0.0645 (2)	0.4121 (2)	0.50562 (5)	0.0185 (3)	
H10'	−0.0580	0.4598	0.5130	0.022*	
C9'	0.2104 (2)	0.3945 (2)	0.54290 (6)	0.0193 (3)	
H9'	0.1857	0.4294	0.5757	0.023*	
C4'	0.5558 (2)	0.2916 (2)	0.56866 (5)	0.0193 (3)	
H4'	0.6804	0.2868	0.5558	0.023*	
C2'	0.5490 (2)	0.2677 (2)	0.61826 (5)	0.0179 (3)	
C3'	0.7287 (2)	0.2047 (2)	0.64785 (5)	0.0186 (3)	
C5'	0.7559 (2)	−0.0070 (2)	0.72222 (6)	0.0220 (3)	
H5A'	0.7835	0.0247	0.7573	0.026*	
H5B'	0.8765	−0.0475	0.7074	0.026*	
C6'	0.5976 (2)	−0.1640 (2)	0.71715 (5)	0.0190 (3)	
H6'	0.5717	−0.2175	0.7502	0.023*	
C7'	0.4137 (2)	−0.0660 (3)	0.69485 (5)	0.0200 (3)	
H7A'	0.3879	−0.1059	0.6603	0.024*	
H7B'	0.3006	−0.0968	0.7142	0.024*	
C7A'	0.4561 (2)	0.1466 (2)	0.69725 (5)	0.0176 (3)	
H7C'	0.4138	0.1998	0.7291	0.021*	
C1'	0.3793 (2)	0.2681 (2)	0.65274 (5)	0.0169 (3)	
H1'	0.2651	0.2069	0.6362	0.020*	
C13'	0.4305 (2)	0.2780 (2)	0.48130 (6)	0.0199 (3)	
H13'	0.5548	0.2371	0.4732	0.024*	
C12'	0.2860 (2)	0.2915 (2)	0.44377 (5)	0.0198 (3)	
H12'	0.3100	0.2577	0.4108	0.024*	

### Atomic displacement parameters (Å^2^)

**Table:**

	*U*^11^	*U*^22^	*U*^33^	*U*^12^	*U*^13^	*U*^23^
O4	0.0276 (6)	0.0393 (8)	0.0296 (6)	0.0024 (6)	0.0041 (4)	0.0055 (6)
O3	0.0216 (5)	0.0263 (6)	0.0200 (5)	0.0046 (5)	0.0061 (4)	0.0038 (5)
O5	0.0488 (7)	0.0514 (9)	0.0208 (6)	−0.0125 (7)	0.0030 (5)	−0.0045 (6)
O2	0.0145 (5)	0.0288 (7)	0.0290 (6)	0.0000 (5)	−0.0015 (4)	−0.0011 (5)
O1	0.0162 (5)	0.0249 (6)	0.0280 (5)	−0.0014 (5)	0.0051 (4)	0.0020 (5)
O4'	0.0290 (6)	0.0305 (7)	0.0233 (5)	−0.0010 (5)	0.0004 (4)	−0.0059 (5)
O3'	0.0184 (5)	0.0207 (6)	0.0247 (5)	0.0009 (5)	−0.0004 (4)	−0.0056 (5)
O5'	0.0194 (5)	0.0293 (7)	0.0272 (5)	0.0044 (5)	0.0025 (4)	0.0036 (5)
O2'	0.0146 (5)	0.0271 (6)	0.0322 (5)	0.0012 (5)	0.0031 (4)	0.0004 (5)
O1'	0.0176 (5)	0.0155 (6)	0.0296 (5)	0.0005 (4)	0.0043 (4)	−0.0030 (5)
N1	0.0135 (5)	0.0232 (7)	0.0204 (6)	0.0020 (6)	0.0018 (4)	−0.0001 (6)
N2'	0.0218 (6)	0.0180 (7)	0.0205 (6)	−0.0023 (6)	0.0027 (5)	0.0034 (5)
C8'	0.0214 (7)	0.0141 (8)	0.0205 (7)	−0.0017 (6)	0.0036 (6)	0.0026 (6)
N1'	0.0153 (6)	0.0176 (7)	0.0215 (6)	0.0003 (5)	−0.0026 (5)	−0.0014 (5)
N2	0.0366 (7)	0.0233 (8)	0.0206 (6)	0.0000 (7)	0.0035 (5)	0.0014 (6)
C11	0.0309 (8)	0.0168 (9)	0.0216 (7)	−0.0010 (7)	0.0046 (6)	−0.0016 (7)
C10	0.0246 (7)	0.0254 (9)	0.0229 (7)	0.0033 (7)	0.0010 (6)	−0.0015 (7)
C9	0.0272 (8)	0.0226 (9)	0.0192 (7)	0.0040 (7)	−0.0011 (6)	−0.0010 (7)
C8	0.0243 (7)	0.0162 (8)	0.0206 (7)	−0.0016 (7)	0.0000 (5)	−0.0024 (6)
C4	0.0184 (7)	0.0185 (9)	0.0238 (7)	0.0003 (6)	−0.0015 (5)	0.0004 (7)
C2	0.0170 (7)	0.0159 (8)	0.0217 (7)	0.0015 (6)	0.0007 (5)	0.0016 (6)
C1	0.0160 (6)	0.0182 (8)	0.0201 (7)	0.0005 (6)	0.0013 (5)	0.0007 (7)
C7A	0.0146 (6)	0.0179 (8)	0.0228 (7)	−0.0010 (6)	0.0003 (5)	0.0001 (6)
C7	0.0146 (6)	0.0249 (9)	0.0204 (7)	0.0008 (6)	0.0005 (5)	0.0005 (7)
C6	0.0190 (7)	0.0207 (8)	0.0194 (7)	0.0010 (7)	0.0018 (5)	−0.0003 (7)
C12	0.0326 (8)	0.0200 (9)	0.0195 (7)	−0.0045 (7)	−0.0043 (6)	0.0007 (7)
C13	0.0231 (7)	0.0187 (8)	0.0251 (7)	−0.0019 (7)	−0.0023 (6)	−0.0003 (7)
C5	0.0169 (7)	0.0283 (9)	0.0210 (7)	0.0003 (7)	0.0047 (5)	−0.0013 (7)
C3	0.0187 (7)	0.0145 (8)	0.0228 (7)	0.0034 (6)	−0.0004 (5)	0.0008 (6)
C11'	0.0200 (7)	0.0140 (8)	0.0214 (7)	−0.0016 (6)	0.0014 (5)	0.0032 (6)
C10'	0.0181 (6)	0.0151 (8)	0.0226 (7)	0.0013 (6)	0.0057 (5)	0.0012 (6)
C9'	0.0226 (7)	0.0166 (8)	0.0192 (7)	−0.0001 (6)	0.0048 (6)	0.0009 (6)
C4'	0.0176 (6)	0.0158 (8)	0.0248 (7)	−0.0009 (6)	0.0056 (6)	−0.0001 (6)
C2'	0.0164 (7)	0.0134 (8)	0.0239 (7)	0.0001 (6)	0.0012 (5)	−0.0005 (6)
C3'	0.0173 (7)	0.0147 (8)	0.0238 (7)	−0.0008 (6)	0.0002 (5)	−0.0035 (7)
C5'	0.0217 (7)	0.0198 (9)	0.0240 (7)	0.0001 (7)	−0.0054 (5)	−0.0009 (7)
C6'	0.0202 (7)	0.0197 (9)	0.0172 (6)	0.0013 (7)	0.0014 (5)	0.0003 (6)
C7'	0.0181 (6)	0.0197 (8)	0.0222 (7)	−0.0012 (7)	−0.0014 (5)	0.0009 (7)
C7A'	0.0150 (6)	0.0200 (9)	0.0179 (7)	0.0002 (6)	0.0008 (5)	−0.0025 (6)
C1'	0.0169 (7)	0.0149 (8)	0.0190 (6)	0.0007 (6)	0.0006 (5)	−0.0032 (6)
C13'	0.0191 (7)	0.0171 (8)	0.0240 (7)	0.0018 (6)	0.0080 (6)	0.0031 (7)
C12'	0.0233 (7)	0.0181 (8)	0.0182 (6)	−0.0003 (7)	0.0052 (6)	0.0004 (6)

### Geometric parameters (Å, º)

**Table:**

O4—N2	1.2262 (18)	C1—C7A	1.5439 (19)
O3—C6	1.414 (2)	C1—H1	0.9800
O3—H3	0.8200	C7A—C7	1.518 (2)
O5—N2	1.2328 (18)	C7A—H7C	0.9800
O2—C3	1.2321 (19)	C7—C6	1.5374 (18)
O1—C1	1.4100 (19)	C7—H7A	0.9700
O1—H1A	0.8200	C7—H7B	0.9700
O4'—N2'	1.2266 (18)	C6—C5	1.5455 (19)
O3'—C6'	1.4316 (19)	C6—H6	0.9800
O3'—H3'	0.8200	C12—C13	1.389 (2)
O5'—N2'	1.2319 (17)	C12—H12	0.9300
O2'—C3'	1.2209 (18)	C13—H13	0.9300
O1'—C1'	1.434 (2)	C5—H5A	0.9700
O1'—H1A'	0.8200	C5—H5B	0.9700
N1—C3	1.3409 (19)	C11'—C12'	1.384 (2)
N1—C5	1.4608 (18)	C11'—C10'	1.391 (2)
N1—C7A	1.4637 (17)	C10'—C9'	1.383 (2)
N2'—C11'	1.4657 (19)	C10'—H10'	0.9300
C8'—C9'	1.400 (2)	C9'—H9'	0.9300
C8'—C13'	1.405 (2)	C4'—C2'	1.334 (2)
C8'—C4'	1.466 (2)	C4'—H4'	0.9300
N1'—C3'	1.3760 (19)	C2'—C3'	1.498 (2)
N1'—C5'	1.461 (2)	C2'—C1'	1.5073 (19)
N1'—C7A'	1.4696 (17)	C5'—C6'	1.549 (2)
N2—C11	1.4655 (19)	C5'—H5A'	0.9700
C11—C10	1.379 (2)	C5'—H5B'	0.9700
C11—C12	1.381 (2)	C6'—C7'	1.531 (2)
C10—C9	1.386 (2)	C6'—H6'	0.9800
C10—H10	0.9300	C7'—C7A'	1.526 (2)
C9—C8	1.402 (2)	C7'—H7A'	0.9700
C9—H9	0.9300	C7'—H7B'	0.9700
C8—C13	1.396 (2)	C7A'—C1'	1.537 (2)
C8—C4	1.467 (2)	C7A'—H7C'	0.9800
C4—C2	1.333 (2)	C1'—H1'	0.9800
C4—H4	0.9300	C13'—C12'	1.379 (2)
C2—C3	1.497 (2)	C13'—H13'	0.9300
C2—C1	1.5236 (19)	C12'—H12'	0.9300
			
C6—O3—H3	109.5	C12—C13—H13	119.2
C1—O1—H1A	109.5	C8—C13—H13	119.2
C6'—O3'—H3'	109.5	N1—C5—C6	102.60 (11)
C1'—O1'—H1A'	109.5	N1—C5—H5A	111.2
C3—N1—C5	129.22 (13)	C6—C5—H5A	111.2
C3—N1—C7A	114.76 (11)	N1—C5—H5B	111.2
C5—N1—C7A	113.38 (11)	C6—C5—H5B	111.2
O4'—N2'—O5'	123.76 (13)	H5A—C5—H5B	109.2
O4'—N2'—C11'	118.14 (12)	O2—C3—N1	125.74 (13)
O5'—N2'—C11'	118.08 (12)	O2—C3—C2	127.10 (13)
C9'—C8'—C13'	118.70 (13)	N1—C3—C2	107.10 (12)
C9'—C8'—C4'	124.39 (13)	C12'—C11'—C10'	122.56 (14)
C13'—C8'—C4'	116.89 (13)	C12'—C11'—N2'	118.47 (13)
C3'—N1'—C5'	121.79 (13)	C10'—C11'—N2'	118.92 (13)
C3'—N1'—C7A'	111.93 (11)	C9'—C10'—C11'	118.88 (13)
C5'—N1'—C7A'	109.02 (12)	C9'—C10'—H10'	120.6
O4—N2—O5	123.54 (13)	C11'—C10'—H10'	120.6
O4—N2—C11	118.90 (12)	C10'—C9'—C8'	120.32 (14)
O5—N2—C11	117.55 (13)	C10'—C9'—H9'	119.8
C10—C11—C12	122.37 (14)	C8'—C9'—H9'	119.8
C10—C11—N2	119.32 (14)	C2'—C4'—C8'	129.29 (14)
C12—C11—N2	118.31 (13)	C2'—C4'—H4'	115.4
C11—C10—C9	119.04 (15)	C8'—C4'—H4'	115.4
C11—C10—H10	120.5	C4'—C2'—C3'	119.78 (13)
C9—C10—H10	120.5	C4'—C2'—C1'	131.40 (13)
C10—C9—C8	120.56 (14)	C3'—C2'—C1'	108.31 (12)
C10—C9—H9	119.7	O2'—C3'—N1'	125.71 (13)
C8—C9—H9	119.7	O2'—C3'—C2'	127.20 (13)
C13—C8—C9	118.40 (14)	N1'—C3'—C2'	107.07 (12)
C13—C8—C4	117.06 (13)	N1'—C5'—C6'	104.55 (11)
C9—C8—C4	124.50 (13)	N1'—C5'—H5A'	110.8
C2—C4—C8	131.99 (14)	C6'—C5'—H5A'	110.8
C2—C4—H4	114.0	N1'—C5'—H5B'	110.8
C8—C4—H4	114.0	C6'—C5'—H5B'	110.8
C4—C2—C3	118.93 (13)	H5A'—C5'—H5B'	108.9
C4—C2—C1	133.59 (13)	O3'—C6'—C7'	107.72 (12)
C3—C2—C1	107.43 (11)	O3'—C6'—C5'	112.42 (12)
O1—C1—C2	114.00 (12)	C7'—C6'—C5'	105.71 (13)
O1—C1—C7A	111.46 (13)	O3'—C6'—H6'	110.3
C2—C1—C7A	102.77 (11)	C7'—C6'—H6'	110.3
O1—C1—H1	109.5	C5'—C6'—H6'	110.3
C2—C1—H1	109.5	C7A'—C7'—C6'	105.95 (12)
C7A—C1—H1	109.5	C7A'—C7'—H7A'	110.5
N1—C7A—C7	102.11 (11)	C6'—C7'—H7A'	110.5
N1—C7A—C1	103.90 (11)	C7A'—C7'—H7B'	110.5
C7—C7A—C1	121.27 (13)	C6'—C7'—H7B'	110.5
N1—C7A—H7C	109.6	H7A'—C7'—H7B'	108.7
C7—C7A—H7C	109.6	N1'—C7A'—C7'	103.06 (12)
C1—C7A—H7C	109.6	N1'—C7A'—C1'	104.75 (11)
C7A—C7—C6	102.61 (11)	C7'—C7A'—C1'	117.14 (12)
C7A—C7—H7A	111.2	N1'—C7A'—H7C'	110.5
C6—C7—H7A	111.2	C7'—C7A'—H7C'	110.5
C7A—C7—H7B	111.2	C1'—C7A'—H7C'	110.5
C6—C7—H7B	111.2	O1'—C1'—C2'	111.62 (12)
H7A—C7—H7B	109.2	O1'—C1'—C7A'	112.18 (11)
O3—C6—C5	113.29 (13)	C2'—C1'—C7A'	102.81 (11)
O3—C6—C7	110.09 (12)	O1'—C1'—H1'	110.0
C5—C6—C7	103.96 (11)	C2'—C1'—H1'	110.0
O3—C6—H6	109.8	C7A'—C1'—H1'	110.0
C5—C6—H6	109.8	C12'—C13'—C8'	121.81 (13)
C7—C6—H6	109.8	C12'—C13'—H13'	119.1
C11—C12—C13	117.93 (14)	C8'—C13'—H13'	119.1
C11—C12—H12	121.0	C13'—C12'—C11'	117.65 (13)
C13—C12—H12	121.0	C13'—C12'—H12'	121.2
C12—C13—C8	121.61 (14)	C11'—C12'—H12'	121.2
			
O4—N2—C11—C10	26.1 (2)	O4'—N2'—C11'—C12'	11.0 (2)
O5—N2—C11—C10	−154.96 (17)	O5'—N2'—C11'—C12'	−170.61 (15)
O4—N2—C11—C12	−153.41 (17)	O4'—N2'—C11'—C10'	−166.52 (15)
O5—N2—C11—C12	25.5 (2)	O5'—N2'—C11'—C10'	11.9 (2)
C12—C11—C10—C9	1.3 (3)	C12'—C11'—C10'—C9'	−2.3 (2)
N2—C11—C10—C9	−178.18 (16)	N2'—C11'—C10'—C9'	175.13 (14)
C11—C10—C9—C8	1.6 (3)	C11'—C10'—C9'—C8'	0.7 (2)
C10—C9—C8—C13	−3.3 (3)	C13'—C8'—C9'—C10'	1.8 (2)
C10—C9—C8—C4	179.20 (16)	C4'—C8'—C9'—C10'	−176.78 (15)
C13—C8—C4—C2	165.53 (19)	C9'—C8'—C4'—C2'	20.8 (3)
C9—C8—C4—C2	−16.9 (3)	C13'—C8'—C4'—C2'	−157.82 (17)
C8—C4—C2—C3	177.05 (17)	C8'—C4'—C2'—C3'	170.07 (16)
C8—C4—C2—C1	0.0 (3)	C8'—C4'—C2'—C1'	−0.8 (3)
C4—C2—C1—O1	−43.3 (3)	C5'—N1'—C3'—O2'	−36.3 (2)
C3—C2—C1—O1	139.45 (13)	C7A'—N1'—C3'—O2'	−167.84 (16)
C4—C2—C1—C7A	−164.06 (19)	C5'—N1'—C3'—C2'	141.93 (13)
C3—C2—C1—C7A	18.68 (16)	C7A'—N1'—C3'—C2'	10.41 (17)
C3—N1—C7A—C7	140.96 (14)	C4'—C2'—C3'—O2'	10.2 (3)
C5—N1—C7A—C7	−22.39 (18)	C1'—C2'—C3'—O2'	−177.00 (16)
C3—N1—C7A—C1	14.10 (18)	C4'—C2'—C3'—N1'	−168.00 (15)
C5—N1—C7A—C1	−149.25 (14)	C1'—C2'—C3'—N1'	4.77 (17)
O1—C1—C7A—N1	−141.64 (12)	C3'—N1'—C5'—C6'	−103.79 (15)
C2—C1—C7A—N1	−19.14 (16)	C7A'—N1'—C5'—C6'	28.93 (15)
O1—C1—C7A—C7	104.60 (16)	N1'—C5'—C6'—O3'	107.05 (14)
C2—C1—C7A—C7	−132.91 (14)	N1'—C5'—C6'—C7'	−10.21 (15)
N1—C7A—C7—C6	36.32 (15)	O3'—C6'—C7'—C7A'	−131.16 (13)
C1—C7A—C7—C6	151.00 (14)	C5'—C6'—C7'—C7A'	−10.78 (15)
C7A—C7—C6—O3	−159.78 (13)	C3'—N1'—C7A'—C7'	102.01 (14)
C7A—C7—C6—C5	−38.13 (16)	C5'—N1'—C7A'—C7'	−35.68 (14)
C10—C11—C12—C13	−2.3 (3)	C3'—N1'—C7A'—C1'	−21.04 (17)
N2—C11—C12—C13	177.18 (16)	C5'—N1'—C7A'—C1'	−158.73 (12)
C11—C12—C13—C8	0.5 (3)	C6'—C7'—C7A'—N1'	27.53 (14)
C9—C8—C13—C12	2.2 (3)	C6'—C7'—C7A'—C1'	141.91 (12)
C4—C8—C13—C12	179.95 (16)	C4'—C2'—C1'—O1'	−84.7 (2)
C3—N1—C5—C6	−161.69 (16)	C3'—C2'—C1'—O1'	103.71 (14)
C7A—N1—C5—C6	−1.32 (18)	C4'—C2'—C1'—C7A'	154.90 (17)
O3—C6—C5—N1	143.86 (13)	C3'—C2'—C1'—C7A'	−16.73 (15)
C7—C6—C5—N1	24.36 (16)	N1'—C7A'—C1'—O1'	−97.99 (13)
C5—N1—C3—O2	−19.5 (3)	C7'—C7A'—C1'—O1'	148.58 (12)
C7A—N1—C3—O2	−179.69 (15)	N1'—C7A'—C1'—C2'	22.06 (15)
C5—N1—C3—C2	157.98 (16)	C7'—C7A'—C1'—C2'	−91.37 (14)
C7A—N1—C3—C2	−2.17 (18)	C9'—C8'—C13'—C12'	−3.0 (2)
C4—C2—C3—O2	−11.3 (3)	C4'—C8'—C13'—C12'	175.74 (15)
C1—C2—C3—O2	166.43 (16)	C8'—C13'—C12'—C11'	1.5 (2)
C4—C2—C3—N1	171.22 (16)	C10'—C11'—C12'—C13'	1.2 (2)
C1—C2—C3—N1	−11.05 (17)	N2'—C11'—C12'—C13'	−176.23 (14)

### Hydrogen-bond geometry (Å, º)

**Table:**

*D*—H···*A*	*D*—H	H···*A*	*D*···*A*	*D*—H···*A*
O3—H3···O1′^i^	0.82	1.93	2.7303 (14)	166
O1—H1*A*···O2^ii^	0.82	1.89	2.6993 (15)	169
O3′—H3′···O3^iii^	0.82	1.95	2.7585 (15)	171
O1′—H1*A*′···O3′^iv^	0.82	1.93	2.7451 (15)	174
C1′—H1′···O2′^ii^	0.98	2.52	3.3655 (17)	144
C1′—H1′···O5′^v^	0.98	2.55	3.3242 (19)	136
C10—H10···O1^vi^	0.93	2.54	3.126 (2)	121
C12′—H12′···O3′^vii^	0.93	2.57	3.5014 (18)	174
C13—H13···O4^i^	0.93	2.51	3.2644 (19)	138
C13′—H13′···O5′^i^	0.93	2.51	3.1160 (18)	123

Symmetry codes: (i) *x*+1, *y*, *z*; (ii) *x*−1, *y*, *z*; (iii) *x*, *y*−1, *z*; (iv) *x*, *y*+1, *z*; (v) −*x*, *y*−1/2, −*z*+1; (vi) −*x*+1, *y*+1/2, −*z*+2; (vii) −*x*+1, *y*+1/2, −*z*+1.
